# Determining the amount of waste plastics in the feed of Austrian waste-to-energy facilities

**DOI:** 10.1177/0734242X16660372

**Published:** 2016-07-29

**Authors:** Therese Schwarzböck, Emile Van Eygen, Helmut Rechberger, Johann Fellner

**Affiliations:** 1 Institute for Water Quality, Resource & Waste Management, TU Wien, Vienna, Austria; 2 Christian Doppler Laboratory for Anthropogenic Resources, TU Wien, Vienna, Austria

**Keywords:** Plastics waste generation, plastic content, thermal utilisation, waste-to-energy, waste incineration, Balance Method, municipal solid waste

## Abstract

Although thermal recovery of waste plastics is widely practiced in many European countries, reliable information on the amount of waste plastics in the feed of waste-to-energy plants is rare. In most cases the amount of plastics present in commingled waste, such as municipal solid waste, commercial, or industrial waste, is estimated based on a few waste sorting campaigns, which are of limited significance with regard to the characterisation of plastic flows. In the present study, an alternative approach, the so-called Balance Method, is used to determine the total amount of plastics thermally recovered in Austria’s waste incineration facilities in 2014. The results indicate that the plastics content in the waste feed may vary considerably among different plants but also over time. Monthly averages determined range between 8 and 26 wt% of waste plastics. The study reveals an average waste plastics content in the feed of Austria’s waste-to-energy plants of 16.5 wt%, which is considerably above findings from sorting campaigns conducted in Austria. In total, about 385 kt of waste plastics were thermally recovered in all Austrian waste-to-energy plants in 2014, which equals to 45 kg plastics cap^-1^. In addition, the amount of plastics co-combusted in industrial plants yields a total thermal utilisation rate of 70 kg cap^-1^ a^-1^ for Austria. This is significantly above published rates, for example, in Germany reported rates for 2013 are in the range of only 40 kg of waste plastics combusted per capita.

## Introduction

Consumption of plastics and thus also the generation of waste plastics has increased tremendously during the last decades. Whereas at the beginning of the 1980s global consumption of plastics amounted to about 65m t, in 2014 worldwide production has increased to more than 300m t (PlasticsEurope, 2015). A significant share of plastics (more than one-third) is used in short-life products, such as packaging (PlasticsEurope, 2015). These plastics almost directly contribute to present waste generation, whereas plastics used in other sectors, such as the building and construction sector (e.g. pipes, flooring) or the automotive sector, will become wastes with a delay of some years to decades.

To deal with the increasing waste plastics quantities, appropriate waste management systems have to be set up, as the European Commission is pushing for increased recycling rates, among others, for various materials in packaging waste including plastics (European Commission, 2015). However, there is a need for detailed knowledge on the current situation of material flows and stocks to understand what potential for recycling is available. For metals, this kind of information is quite well established, but for plastics, only limited data is available on the flows through society (Van Eygen et al., submitted). A challenge therefore still remains in accurately recording the plastics flows in waste production and waste treatment.

Many affluent countries have introduced separate collection of certain plastic wastes (with special focus on packaging plastics) in recent years. The aim is, on the one hand, to reduce the quantity of mixed household wastes and, on the other hand, to generate post-consumer waste streams that contain one type of polymer only. The latter is understood to be a prerequisite for high-quality recycling of plastics waste.

The amount of plastics waste separately collected is typically recorded quite accurately, owing to its economic value and its positive image for the plastics industry, as recycling separately collected post-consumer plastics may at least partly allow closing material cycles and is thus considered an important process for the plastics industry to contribute to resource conservation. There is for instance a 40% to 90% reduction in energy consumption by producing recycled plastic compared with producing plastic from virgin materials (oil and gas) (Association of Cities and Regions for Recycling, 2004).

Whereas, the amount of plastics recycled after separate collection can be monitored quite easily (it equals the total output of recycling facilities for plastic waste plus all plastic waste exported for recycling, whereby for the latter a realistic material recovery rate has to be assumed), the quantification of the total plastic waste generated at a regional or nationwide level is rather difficult. This is because the vast majority of plastics is collected in waste streams, such as municipal solid waste (MSW), commercial waste (CW) or industrial waste (IW), where they are commingled with various other materials.

Thus, total plastic waste generation $P_{tot}$ is usually estimated as:

(1) $$P_{tot}=\overset{n}{\underset{i=1}{\sum }}W_{i}\cdot c_{pi}$$

where statistics on the generation of different wastes $W_{i}$ are combined with data on the waste composition, in particular the content of plastics $c_{pi}$ Information on the latter is either close to 100% for separately collected waste plastics, derived from literature (e.g. plastics content in end-of-life vehicles), or can be based on sorting analyses for different mixed waste types. These latter analyses are at most conducted once a year, but owing to the fact that waste composition may show significant variations even over time periods of a few days (Morf and Brunner, 1998; Obermoser et al., 2009), a few single sorting campaigns are not sufficient to calculate a reliable annual average plastic content. In addition, some types of waste streams, such as IW, but also CW, may show distinctly different compositions with respect to their contents of plastics, depending on the activities of the respective companies generating the waste. Furthermore, the plastic content of wastes determined via sorting analyses may be of limited significance even for the respective waste sample analysed: (1) owing to the lack of visual recognisability of different materials (e.g. synthetic versus biogenic fibres) and (2) owing to the fact that waste sorting analyses are typically aimed at determining the content of different aggregated waste fractions (such as biowaste, hygienic products, composite materials, etc.) that do not necessarily contain only plastics or are free of plastics (Dahlén and Lagerkvist, 2008). Thus, results of sorting analyses further require the determination/assignment of average plastic contents to the single waste fractions sorted out.

All these limitations demonstrate that figures for total quantities of plastic wastes generated are associated with significant uncertainties. Hence, also data on the recycling or thermal recovery quota of plastic waste, as published for different European countries (e.g. BIO Intelligence Service, 2013; Bogucka et al., 2008; PlasticsEurope, 2015) are rather unclear.

Therefore, a significant share of waste plastics is collected via ‘mixed’ wastes (MSW, CW, and IW), and plastic contents of these waste are relatively uncertain. The aim of the current study is thus to determine the total amount of waste plastics present in these wastes. The investigations are conducted for Austria for the year 2014. Austria is chosen as a case study, as there is a landfill ban for wastes with more than 50 g organic carbon per kilogram dry waste, meaning that mixed wastes containing even minor amounts of plastics are to be diverted into thermal treatment plants. For the determination of the plastics content in the incinerated commingled wastes, an alternative approach to sorting analyses, the so-called Balance Method (according to Fellner et al., 2007), is applied. This method was originally developed to evaluate the ratio of energy from biogenic and thus renewables sources in the feed of waste-to-energy (WtE) plants, but also allows calculating the content of plastics (fossil material) in the waste feed.

## Materials and methods

### Balance Method

**Figure 1.**
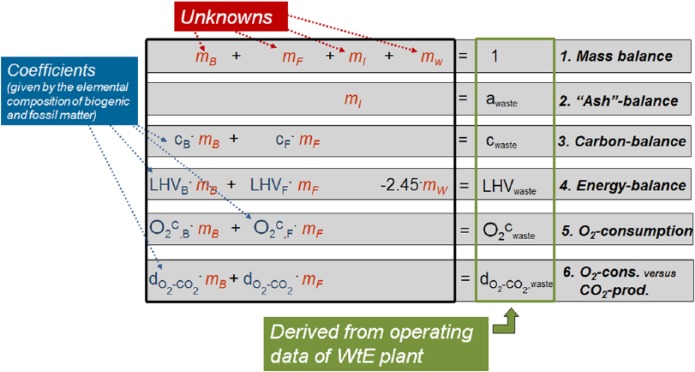
Simplified set of equations used by the Balance Method (based on Staber et al., 2008); the left side of the equations represent the theoretical balance (utilising information on the elemental composition of biogenic and fossil organic matter) that has to be attuned to the different waste characteristics derived from operation data of the WtE plant (right side of the equations). WtE: waste-to-energy.

The Balance Method, applied in the present study to determine the content of plastics in mixed wastes, combines data on the elemental composition of moisture- and ash-free (maaf) biogenic and fossil organic matter with routinely measured operating data of the WtE plant. In principle, the method utilises one energy balance and five mass balances, whereby each balance describes a certain waste characteristic (e.g. content of organic carbon, lower calorific value, ash content). Each balance equation encompasses a theoretically derived term (left side of equations) that has to be attuned to measured data of the incineration plant (right side of equations). A simplified structure of the set of equations is illustrated in Figure 1. A detailed mathematical description of each equation is given in Fellner et al. (2007).

**Figure 2.**
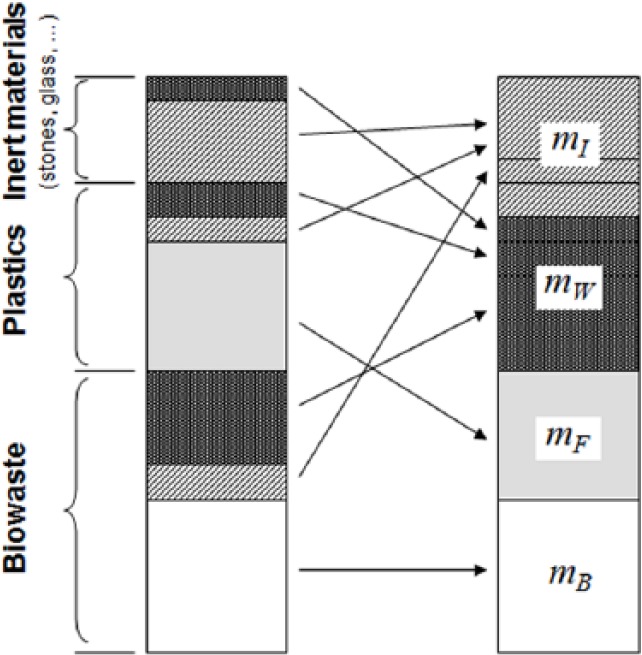
Split-up of waste fractions into the four ‘material groups’ (*m_B_, m_F_, m_W_*, and *m_I_*), which represent the unknowns in the set of six equations (based on Fellner et al., 2007).

**Figure 3.**
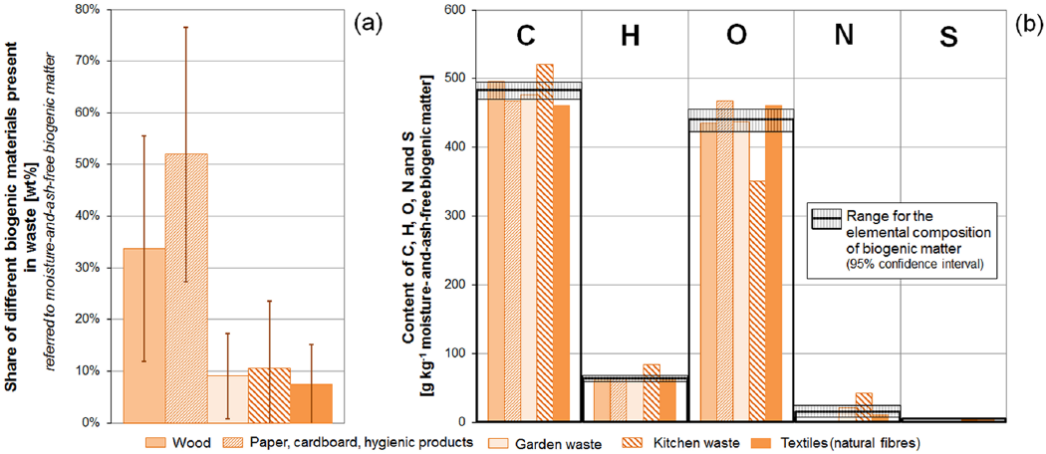
(a) Possible ratios of different biogenic compounds (e.g. wood, paper, etc.) present in mixed wastes (referred to maaf biogenic matter); (b) elemental composition (content of carbon, hydrogen, oxygen, nitrogen, and sulphur) of different biogenic materials present in mixed waste, including an estimate for the range of the elemental composition (indicated as hatched area) of biogenic matter present in mixed waste (referred to maaf biogenic matter).

For setting up the six balance equations, the waste mass is virtually divided into four ‘material groups’: inert (*m_I_*), biogenic and fossil organic materials (*m_B_, m_F_*), and water (*m_W_*) (Figure 2). Inert materials include all incombustible solid residues like glass, stones, ashes, or other inorganic matter from biowaste and plastics (e.g. kaolin in paper or inorganic additives in plastics). Biogenic and fossil organic material groups refer only to the maaf organic matter (see Figure 2). As the qualitative composition of organic materials in mixed wastes is usually well known (e.g. biogenic matter encompasses paper, wood, kitchen waste, etc. and fossil organic matter includes polymers, such as polyethylene (PE), polypropylene (PP), polyethylene terephthalate (PET), polyvinylchloride (PVC), etc.) the content of carbon, hydrogen, oxygen, nitrogen, sulphur, and chlorine of the maaf biogenic and fossil organic materials (*m_B_* and *m_F_*) can be derived. The quantitative shares of the different compounds in the biogenic fraction are of minor significance for the determination of the chemical composition, as the elemental composition of the different biogenic materials present in waste is quite similar and differs only slightly (see Figure 3(b)).

The input data required for the Balance Method comprise information on the elemental composition of maaf biogenic and fossil organic matter present in the waste feed, information on the quantity of fuels incinerated (waste mass and auxiliary fuels), the amount of solid residues and steam produced, as well as data on the volume and composition (O_2_ and CO_2_ content) of the dry flue gas (a graphical overview of the required input data is presented in the supplementary material Figure A.1, available online). For each parameter a respective uncertainty is specified.

Because the system of equations (set of constraints) used within the Balance Method is over-determined (six equations for four unknowns), data reconciliation has to be performed to eliminate data contradiction and to improve the accuracy of the results. The reconciled values are subsequently used to compute the unknown quantities (*m_B_, m_F_, m_W_*, and *m_I_*) including their uncertainties.

The fossil mass fraction *m_F_* represents the content of synthetic polymers in the waste feed of the plant. By considering typical values for the ash content of plastics $a_{p}$ (representing the content of inorganic additives and fillers) the fraction of plastics $c_{P}$ in the waste feed can easily be determined according to:

(2) $$c_{P}=\frac{m_{F}}{\left(1-a_{p}\right)}$$

Based on national material flow studies focusing on Austrian plastics production and consumption (Bogucka and Brunner, 2007; Fehringer and Brunner, 1997), the average content of inorganic additives and fillers $a_{p}$ is estimated to 90 ±40 g kg^-1^ plastics, which is used in the present study.

Prior to solving the set of equations for calculating the mass fraction *m_F_*, the input data (operating data of the WtE plant) are checked regarding their plausibility. Thereto, existing correlations between the flue gas and its composition and the steam production are used (e.g. during the combustion of organic matter the consumption of 1 mole of oxygen gas corresponds to an energy generation of 360 to 400 kJ; and the combustion of 1 g organic carbon produces a heat amount of 34 up to a maximum of 44 kJ) (for details see Fellner et al., 2007). The calculations according to the Balance Method are only performed with plausible data, whereby the temporal resolution of the data used is preferably in the range of hourly averages for most input data.

The analysis algorithm of the Balance Method, including the plausibility check of the input data, has been implemented into the software BIOMA (http://iwr.tuwien.ac.at/ressourcen/downloads/bioma.html), which allows determining the composition of the waste feed with respect to its content of biomass and fossil organic matter. In the frame of the presented study, all analyses according to the Balance Method have been conducted using BIOMA.

### Elemental composition of biogenic and fossil organic matter used for the Balance Method

In principle, the Balance Method is based on the distinct chemical composition (content of carbon, hydrogen, oxygen, nitrogen, sulphur, and chlorine) of maaf biogenic and fossil organic matter and on the differences in the composition of the two materials. As at least qualitative information on the different biogenic and fossil materials present in mixed waste is available (from literature or sorting analyses), plausible ranges for the chemical composition can be derived.

**Figure 4.**
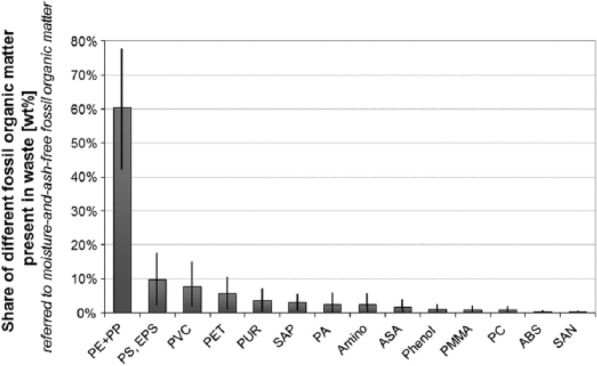
Possible ratios of different plastics (fossil organic) materials (e.g. PE, PVC, PET, etc.) present in mixed wastes (referred to maaf fossil organic matter) – no differentiation between PE and PP was made since their elemental composition is identical. ABS: acrylonitrile butadiene styrene; ASA: acrylonitrile styrene acrylate; EPS: expanded polystyrene; PA: polyamide; PC: polycarbonate; PE: polyethylene; PET: polyethylene terephtalate; PMMA: Poly(methyl methacrylate); PP: polypropylene; PS: polystyrene; PUR: polyurethane; PVC: polyvinyl chloride; SAN: styrene acrylonitrile; SAP: superabsorbent polymers.

Biogenic matter in waste is basically composed of paper, cardboard, hygienic articles, wood, kitchen waste, garden waste, and textiles (natural fibres) (Figure 3(a)), whereas fossil organic matter (plastics) in mixed wastes may include the whole range of different polymers produced (e.g. PE, PP, PET, PVC, polystyrene PS, polyamide PA) (Figure 4).

Combining contents of carbon, hydrogen, oxygen, nitrogen, sulphur, and chlorine in each maaf biogenic or fossil compound (such as wood, paper, or PE) with information on their relative shares (referred to the total amount of maaf biogenic and fossil organic matter, respectively), allows calculating ranges for the elemental composition (content of carbon, hydrogen, oxygen, nitrogen, sulphur, and chlorine) of maaf biogenic and fossil organic matter (see Figure 3(b)). In practise, this has been accomplished by applying the Monte Carlo simulation to the following equations, which are exemplary given for carbon:

(3) $$C_{Bi}=\frac{\sum_{k=1}^{k=n}C_{Bki}\cdot f_{Bki}}{\sum_{j=1}^{k=n}f_{Bki}}$$

(4) $$C_{Fi}=\frac{\sum_{k=1}^{k=n}C_{Fki}\cdot f_{Fki}}{\sum_{j=1}^{k=n}f_{Fki}}$$

where $C_{Bi},C_{Fi}$ is the carbon content in maaf biogenic matter (*fossil organic matter*) present in mixed wastes for simulation run i; $C_{Bki},C_{Fki}$ is the carbon content in maaf biogenic matter (*fossil organic matter*) of compound k (e.g. wood, kitchen waste) for simulation run i, and $f_{Bki},\;f_{Fki}$ is the relative share of maaf biogenic compound k (e.g. wood, kitchen waste) referred to total maaf biogenic matter (*fossil organic matter*) present in mixed wastes for simulation run i

In Figure 3 the data used for determining the elemental composition of maaf biogenic matter present in mixed wastes are given. The respective results of the Monte Carlo simulation are indicated by hatched areas in Figure 3(b) and denote the likely range for the elemental composition. A comparison of the different biogenic compounds present in mixed wastes (wood, paper, garden waste, etc.) demonstrates that they only differ slightly in their elemental composition (particularly in their carbon, hydrogen, and oxygen contents). This finding indicates that the elemental composition of maaf biogenic matter in wastes may vary almost independently from the shares of the biogenic compounds. A probable range for the elemental composition of maaf biogenic organic matter can be derived (see Table 1).

**Table 1.** Elemental composition of maaf biogenic and fossil organic matter present in commingled wastes.

Moisture- and ash-free		Biogenic matter^a^		Fossil matter^a^	
Content of	Unit	Average	SD^b^	Average	SD^b^
C	g kg^-1^	483	9	777	32
H	g kg^-1^	65	2.4	112	11
O	g kg^-1^	443	14	61	26
N^c^	g kg^-1^	7	5	14	11
S^c^	g kg^-1^	1.1	0.5	3	1
Cl^c^	g kg^-1^	−^d^	−	32	24

a Minor differences in the elemental composition compared with values given in Fellner et al. (2007) are owing to an updated database, which considers recent results of waste composition studies.

b 95% confidence interval.

c Contents of N, S and Cl are of minor significance for the results of the Balance Method (see Fellner et al., 2007).

d Cl-content <5 g kg^-1^. (Kost, 2001; LfU, 2003).

SD: standard deviation; C: carbon; H: hydrogen; O: oxygen; N: nitrogen; S: sulphur; Cl: chlorine.

Figure 4 illustrates the assumed composition of waste plastics (with respect to polymers) present in commingled waste. The composition (including the potential range for the different polymers – indicated by the black errors bars) has been derived from polymer consumption statistics (PlasticsEurope, 2010), taking into account the life time of plastics in different applications as well as the potential collection and disposal routes. In addition, data from detailed sorting campaigns conducted in Austria have been considered (Nelles, 1998; TB Hauer, 1999, 2002).

In analogy to the biogenic matter, combining the data given in Figure 4 with the elemental composition of each polymer allows calculating the most likely carbon, hydrogen, oxygen, nitrogen, sulphur, and chlorine content of maaf fossil organic matter present in mixed wastes. Thereto the Monte Carlo simulation (using equation (4)) has been applied. Using the final result of these simulations, a probable range for the elemental composition of maaf biogenic and fossil organic matter is summarised in Table 1.

### Investigated WtE plants in Austria

The feed of 10 Austrian WtE plants, which represent 91% of the waste incinerated in Austria in 2014, has been investigated with respect to its plastic content. Three facilities could not be included in the study as they did not provide all operating data required for the Balance Method or were under reconstruction in the respective time period.

Table 2 gives an overview of the 10 Austrian waste incineration plants investigated. The overall capacity of these facilities amounts to about 2.3m t of waste per year (BMLFUW, 2011). The WtE plants utilise different types of combustion technologies (grate incineration or fluidised bed combustion) and mainly incinerate MSW, CW and IW, sewage sludge, and refuse derived fuels (see Table 2), whereby the share of the different wastes may vary significantly during the investigated time period of one year.

**Table 2.** Overview of the investigated WtE plants in Austria.

WtE plant	Combustion technology	Waste incinerated (qualitative information)
A	Grate incinerator	MSW
B	Grate incinerator	MSW and CW&IW
C	Stationary fluidised bed combustion	RDF and SS
D	Stationary fluidised bed combustion	RDF and SS
E	Circulating fluidised bed combustion	RDF and SS
F	Grate incinerator	CW&IW, and minor amounts of MSW
G	Stationary fluidised bed combustion	RDF, and minor amounts of SS
H	Grate incinerator	MSW, CW&IW, and minor amounts of SS
I	Grate incinerator	MSW
J	Grate incinerator	MSW, CW&IW, and minor amounts of SS

CW&IW: commercial and industrial waste; MSW: municipal solid waste; RDF: refuse derived fuels; SS: sewage sludge; WtE: waste-to-energy.

The Balance Method is applied to the operating data of the 10 WtE plants for a period of 12 months. As for one facility (WtE plant D) the required operating data are incomplete or connected with significant measurement errors, the analysis for this particular plant is reduced to 7 months where reasonable (plausible) operating data are available. The procedure for the application of the Balance Method to the operating data of the WtE plants is described in detail in Schwarzböck et al. (2016). Based on the calculated composition of the waste feed of each plant (using the Balance Method) and their respective annual waste throughput the total amount of waste plastics thermally recovered in 2014 in Austria is determined within the study. The annual average waste plastics content (over all 10 plants) is estimated by summing up the absolute plastics content for all plants (found by applying the calculated plastic content to the actual waste feed) and by relating it to the total amount of waste incinerated in all 10 WtE plants in 2014. To obtain monthly averages considering all 10 plants, the plastics contents per plant are weighted by the monthly waste feed.

## Results and discussion

### Plausibility checks on operating data

An important step prior to determining the waste composition with regards to biomass and fossil organic matter via the Balance Method is the test for plausibility of the operating data. For these tests, operating data are checked for their correlation between flue gas data (expressed as carbon content and oxygen consumption of the waste) and heat production of the plant (in particular lower calorific value of the waste) (example graphs are presented in the supplementary material in Figure B.2 and B.3, available online). The correlation between O_2_ consumption and carbon content is used to finally decide on the plausibility of data points (for details on the plausibility tests see Fellner et al., 2007). Table 3 summarises the share of plausible operating data for all 10 WtE plants that were analysed. With the exception of two plants, all facilities are characterised by a very high share (well above 95%) of plausible operating data. Instable CO_2_ measurements or a lower temporal resolution of the measurements lead to a slightly lower share of plausible operation data for plant D and I, however still above 82% of the data can be used for the analysis. In other words, in total over 96% of the waste feed (thus, almost 2.2m t out of 2.3m t of waste throughput) can be analysed in the study; a sample that can hardly be achieved by any other determination method (such as sorting analysis).

**Table 3.** Share of plausible operating data (given in % of total waste mass combusted) for the WtE plants over a period of 12 months.

WtE plant	A	B	C	D^b^	E	F	G	H	I	J	**Total**
Share of plausible data^a^	98.3	99.3	95.8	84.4	99.5	99.5	98.3	99.0	82.8	99.7	**96.7**

a Expressed as waste mass combusted during the record of plausible operating data referred to in the total waste throughput in per cent.

b Only a period of 7 months has been evaluated.

WtE: waste-to-energy.

### Content of plastics in the feed of Austrian WtE plants

**Figure 5.**
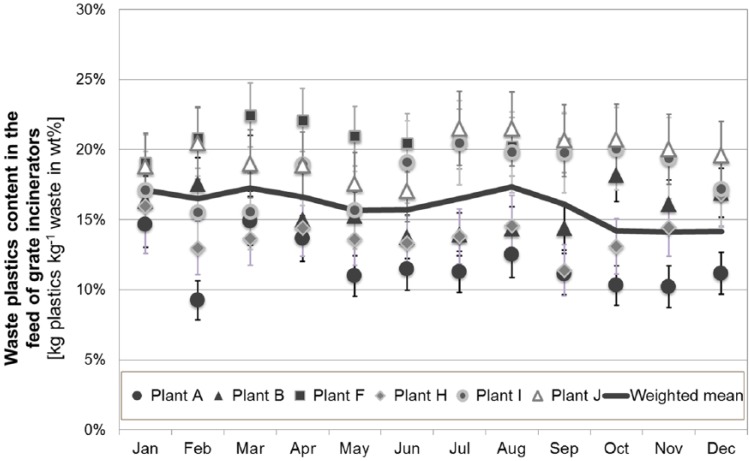
Monthly averages (with standard deviation) of waste plastics content (given in kilograms of waste plastic per kilogram of waste) in the feed of grate incinerators (GI) in Austria, which all mainly utilise MSW, CW, and IW; the monthly mean for all plants (continuous line) accounts for the plastics content and the respective waste mass combusted in each plant.

**Figure 6.**
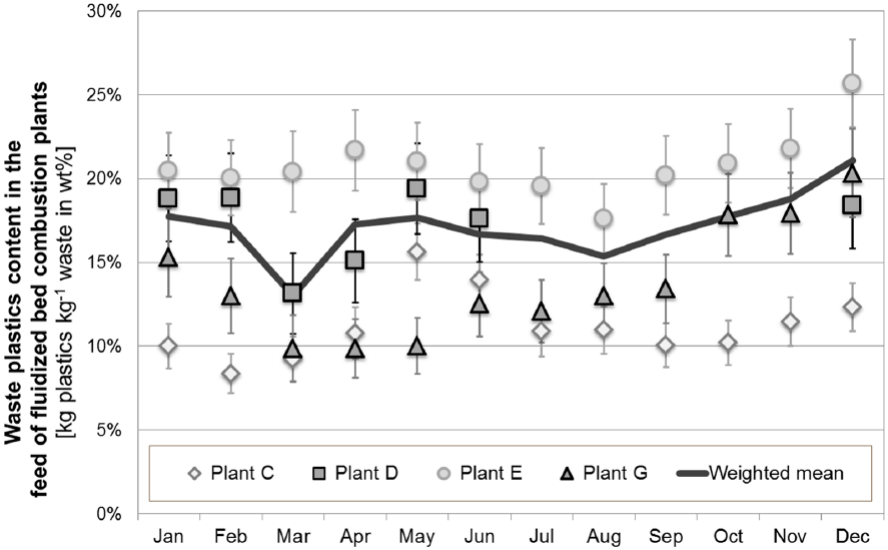
Monthly averages (with standard deviation) of waste plastics content (given in kilograms of waste plastics per kilogram of waste) in the feed of fluidised bed combustion (FBC) plants in Austria, which all mainly utilise refuse derived fuels and sewage sludge; the monthly mean for all plants (continuous line) accounts for the plastics content and the respective waste mass combusted in each plant.

The plausible operating data are subsequently used to analyse the waste composition using the Balance Method. Based thereon the amount of plastics in the waste feed is estimated (according to equation (2)). Figures 5 and 6 summarise and compare the results for the different plants as monthly and as annual averages.

Figure 5 shows the content of waste plastics in the feed of the six WtE plants with grate incineration (GI) that predominantly utilise MSW, CW, and IW. Monthly results for the different WtE plants range from 9 to 23 wt% plastics. This rather large range of plastics content for different plants indicates a regional dependence of the waste feed composition, as almost all plants presented in Figure 5 are situated in different federal states of Austria, which are characterised by different waste collection schemes (e.g. separate collection of either all packaging plastics or just PET bottles). In addition, diverse ratios of commercial, industrial, and MSW may cause significantly different plastic contents in the waste feed. The highest waste plastics contents are observed for plant F, which almost exclusively combusts CW and IW.

Furthermore, temporal variations of waste plastics contents are obvious from Figure 5 as well. For instance, the monthly averages for plant H range from 11 wt% (September) to 17 wt% (December), thereby highlighting that a reliable analysis of the waste composition requires methods that characterise the waste feed over longer time periods, as also concluded in other studies (Fellner et al., 2011; Fuglsang et al., 2014; Obermoser et al., 2009). The annual average waste plastics content in the feed of all Austrian grate incinerators amounts to 16.1 ±1.1 wt%.

For fluidised bed combustion (FBC) plants, observed variations in waste composition (with respect to the contents of waste plastics) are even more pronounced than for grate incinerators (monthly averages for the plastics content in the waste feed range between 8 and 26 wt% – see Figure 6). This finding is somehow unexpected, as the waste utilised at these plants is pre-treated (mechanical separation), which should typically result in more homogenous fuels. However, to the knowledge of the authors, the operation of mechanical treatment plants and also the management of their outputs are both strongly influenced by the fuel demand of the Austrian cement industry, which also utilises significant amounts of refuse derived fuels (RDF). In winter times, cement production in Austria and thus also the demand for high calorific RDF is reduced, which might result in higher amounts of waste plastics being fed into FBC plants. This trend is definitely observable at plant E and plant G, which receive RDF from mechanical waste treatment plants, which also provide fuels for cement kilns. The fuel input into the other FBC plants (C and D) is less influenced by the demand of cement kilns and thus show a less pronounced seasonal trend. For those plants, other factors are expected to have a more dominant influence on the observed trend in waste plastics content in 2014, such as temporary shutdowns (for the revision or renewal) of neighbouring waste combustion plants.

The annual average plastics content in the feed of Austrian fluidised bed combustion plants amounts to 17.3 ±1.2 wt% and is thus slightly above the average content for waste treated in grate incinerators.

**Figure 7.**
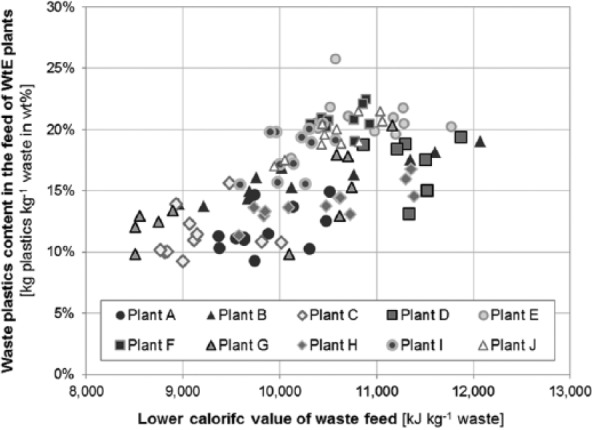
Waste plastics content versus lower calorific value of the waste feed (excluding sewage sludge) for the 10 WtE plants investigated (monthly averages). WtE: waste-to-energy.

When relating the plastics content to the calorific value of the waste (see Figure 7), it becomes evident that an increased energy content of the waste usually goes along with higher contents of plastics. For some WtE plants (e.g. plant B, plant G, plant H, and plant J), a distinct correlation between plastics content and lower calorific value of the waste feed is evident (coefficient of determination higher than 0.6 – not shown in Figure 7); whereas for others, only a general tendency towards more waste plastics in wastes with higher energy contents is observable. For the latter plants it is assumed that other factors, such as the water or ash content of the waste feed, vary more and are hence dominating the calorific value of the waste input.

**Figure 8.**
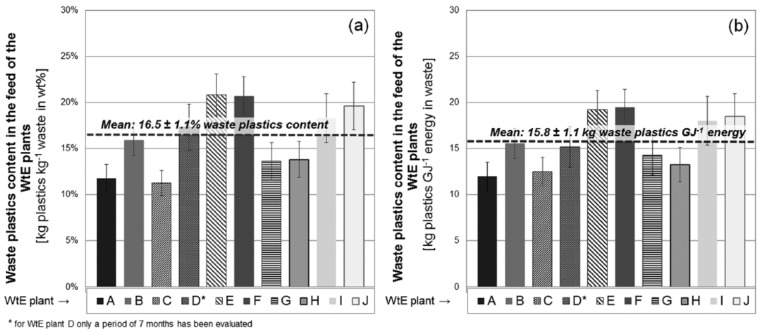
Annual averages (with standard deviation) of waste plastics content in Austrian WtE plants: (a) related to total waste input (in kilograms of plastics per kilogram of waste); (b) related to the calorific value of the solid waste (in kilograms of plastics per GJ of energy content of the waste exclusive sewage sludge). WtE: waste-to-energy.

Annual averages for the plastics contents per kilogram waste and per GJ energy content are presented in Figure 8(a) and (b) for all 10 plants analysed. Again, a high variation of the results for the different plants can be identified, ranging from 11.2 ±1.7 wt% (WtE plant C) to 20.9 ±2.3 wt% (WtE plant E) for the waste mass-related plastics content. The average percentage of plastics in the feed of all Austrian WtE plants amounts to 16.5 ±1.1 wt% (Figure 8(a)).

Calculating energy-related plastics contents (Figure 8(b)), given in kilograms of plastics per GJ calorific value of the waste feed, shows that variations between the input of the different plants are less pronounced. The smallest value of 12.0 kg plastics GJ^-1^ is observed at plant A, whereas plant F shows the highest amount of plastics utilised per unit of energy content (19.5 kg plastics GJ^-1^). The smaller variation of energy-related plastics contents in comparison with the mass-related contents is explained by the fact that variations of the former reflect differing ratios between biogenic and fossil organic matter (plastics) only. Changes in water or ash content, which may obviously also influence the content of plastics, are to some extent already represented by the calorific value of the waste.

Hence, it may be concluded that information on the calorific value of the waste allows better estimates for its content of waste plastics, although possible ranges are still large.

### Total amount of waste plastics thermally utilised in Austria

**Figure 9.**
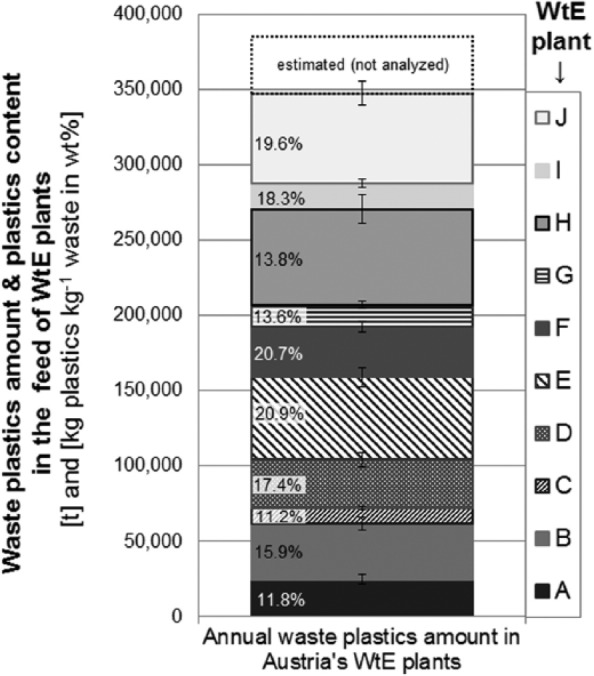
Annual amount of waste plastics in the feed of 10 Austrian WtE plants with an annual waste throughput of approximately 2.3m t (including 0.15m t of sewage sludge).

In Figure 9 the annual flows of waste plastics into all 10 WtE plants are summarised. In total, about 347 ±24 kt of plastics have been thermally utilised in Austria’s waste incineration plants in 2014, neglecting the input into three plants with an annual total waste throughput of about 230 kt (not investigated). Including the waste feed of these three plants into our analysis (assuming an average plastics content of 16.5 ±2.7 wt% in their feed) increases (+38 ±6 kt) the overall amount of waste plastics thermally utilised in Austria to about 385 ±25 kt. Almost 50% of this amount is fed into only three plants (E, H, and J).

The overall energy input via waste plastics into all waste incineration plants totals approximately 14,400 ±900 TJ a^-1^ (assuming an average lower calorific value of waste plastics of 37.5 MJ kg^-1^, according to Kost, 2001), which equals around 11% of Austria’s coal consumption of 130,000 TJ a^-1^ in 2013 (BMWFW, 2015).

## Conclusions

The evaluation of the waste composition of 10 WtE plants in Austria (with a total annual capacity of 2.3m t of waste) via the Balance Method revealed that there are significant differences regarding the plastics content in the waste feed of the different plants. In addition to regional differences, significant temporal variations of the plastics content are observable also at some of the plants analysed. It is assumed that these variations are caused by changing shares of the different types of waste (MSW, CW, IW, RDF), but also by a changing composition of the different wastes combusted. For instance, the plastics content in RDFs utilised in FBC plants may be influenced by the seasonal fuel demand of Austria’s cement kilns, which is definitely lower in winter times. Hence, more plastics may be fed into FBC plants in winter times.

In general, the share of plastics (annual averages) in the waste feed ranges from 11 ±2 to 21 ±3 wt% for the different plants, with an average plastics content of 16.5 wt%. This value is significantly higher than the figures reported by waste sorting studies (8–13 wt%) done in Austria (BMLFUW, 2011; IUT&SDAG, 2014; Schneider and Lebersorger, 2011), which however all focused on MSW and did not account for the plastics present in composites or other mixed material fractions, such as hygienic products. Whereas, the monthly averages of the plastics content determined for plant A (9–15 wt%) are in the range of the results of sorting campaigns, the outcomes for plant I, which also utilises only MSW, indicate a significantly higher plastics content (16–20 wt%). Hence, it can be concluded that there is no typical plastics content in MSW. Furthermore, sorting campaigns most likely tend to underestimate the content of plastics in waste, since even in comparison with results of plant A (waste feed with comparatively low plastics content), sorting analyses generally claim lower contents of plastics in MSW.

The results of the study further indicate a higher monthly variation of the waste plastics content in the feed of FBC plants in comparison with those of GI plants. This might be attributed to the fact that the former are very often designed for specific (industrial) waste streams and that they also compete with industrial co-combustion of wastes (e.g. cement kilns), thereby being exposed to changing fuel demand, which may result in a changing composition of RDFs.

In total about 385 kt of waste plastics have been utilised in Austria’s WtE facilities in 2014, which represents about 45 kg plastics cap^-1^ a^-1^. In addition, the cement industry uses about 210 kt of waste plastics (corresponding to 25 kg cap^-1^ a^-1^) as an alternative fuel (based on data given in Mauschitz, 2015). Results of sorting campaigns (BMLFUW, 2011; IUT&SDAG, 2014; Schneider and Lebersorger, 2011) would reveal a much lower per capita combustion rate of plastics in Austria of 21 to 35 kg plastics cap^-1^ a^-1^ for 2014. In comparison, PlasticsEurope reports 20 kg cap^-1^ a^-1^ of plastics, which were thermally recovered in Europe in 2014 (EU-28 plus Switzerland and Norway) (PlasticsEurope, 2015). This can be easily explained by the fact that within the EU about 40% to 50% of collected waste plastics still ends up at landfills (European Commission, 2015; PlasticsEurope, 2015), whereas in Austria plastic waste is banned from landfilling. However unexpectedly, lower energetic utilisation rates of waste plastics, compared with values determined for Austria, are also reported for Germany, which shows a comparable share of MSW thermally treated (Austria: 37%; Germany: 35% according to Eurostat, 2015). From the figures for 2013 given in Lindner and Hoffmann (2015), per capita incineration rates of 25 kg plastics per year in German WtE plants can be derived, and an additional amount of 15 kg cap^-1^ a^-1^ enters industrial co-combustion plants. Considering the overall waste feed of 21.9m t in 2013 (Confederation of European Waste-to-Energy-Plants, 2013), the average content of plastics in the feed of German WtE plants amounts to only about 10 wt%. This is in the range of results obtained by sorting analyses in Austria (8–13 wt% based on BMLFUW, 2011; IUT&SDAG, 2014; Schneider and Lebersorger, 2011), but is far below the values determined in the present study.

Based on these comparisons of the determined figures with literature values, it can be speculated that plastic flows entering waste incineration facilities are underestimated if they are solely based on results from sorting analyses. Sorting campaigns usually focus on the composition of MSW only and additionally disregard plastics contained in composite material fractions. The herein presented approach (country-wide application of the Balance Method), considers all waste plastics, independent of their compound or combination with other materials. This benefit, together with a very high share of characterised waste in the feed of the WtE facilities (around 91%), the method has to be regarded as the most reliable of all available tools to determine the overall plastics flows that are thermally utilised via waste combustion. Once, the input parameters are collected, the Balance Method allows assessment of the waste composition for any time period of interest (ranging from years to hours) without sampling or supplementary analyses. Only in the case that the WtE plant does not provide CO_2_ measurements for the flue gas, a CO_2_ analyser needs to be installed.

The results of the study are directly incorporated into the national analyses of plastics flows and thus contribute to a better understanding of the recycling potentials of waste plastics in Austria (see Van Eygen et al., submitted).

## Supplementary Material

Supplementary material
