# Supplementary material for: Determining the amount of waste plastics in the feed of Austrian waste-to-energy facilities
Source: Waste Manag Res. 2016 Jul 29;35(2):207–16. doi: 10.1177/0734242X16660372 (PMC5367575; doi:10.1177/0734242X16660372)
Supplement: Supplementary material [file Supplementary_Material.pdf]

Determining the amount of waste plastics in the feed of Austrian Waste to Energy facilities

Supplementary Material

Therese SCHWARZBÖCK[1], Emile VAN EYGEN[2], Helmut RECHBERGER[1], Johann FELLNER[2]

[1] Institute for Water Quality, Resource & Waste Management, TU Wien, Karlsplatz 13, 1040 Vienna, Austria

[2] Christian Doppler Laboratory for Anthropogenic Resources, Institute for Water Quality, Resource & Waste Management, TU Wien, Karlsplatz 13, 1040 Vienna, Austria

A) Summary of the operating data of Waste to Energy plants required as input parameter for the Balance Method

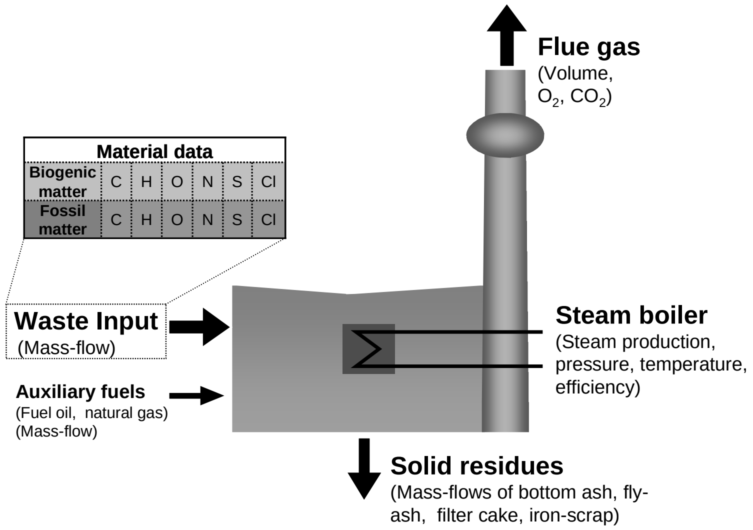

Figure A.1. Required input data for the Balance Method.

B) Examples of plausibility graphs for the Balance Method

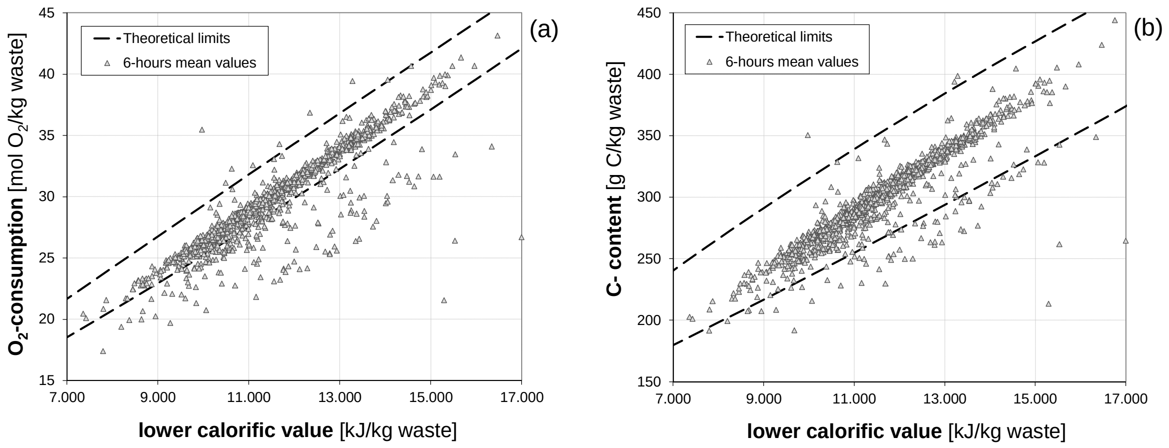

Figure B.2. Results of plausibility checks for operating data of WtE plant B on a 6-hourly basis: (a) Correlation between lower calorific value and O_2 consumption and (b) Correlation between lower calorific value and C content of the waste.

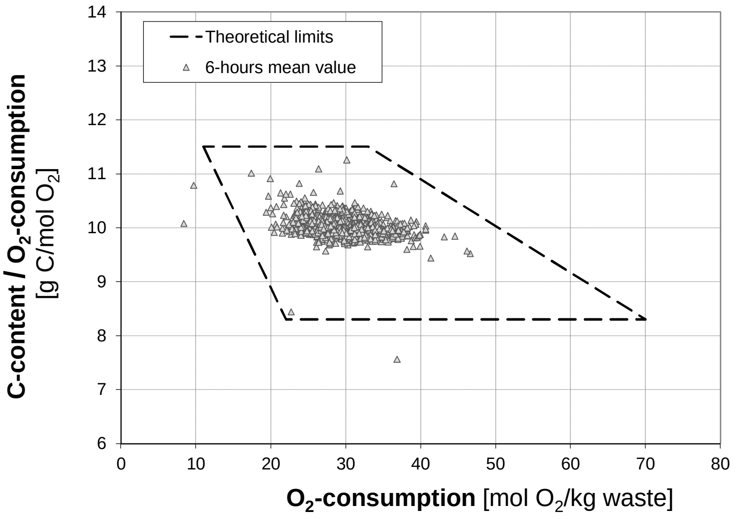

Figure B.3. Results of plausibility checks for the operating data of WtE plant B on a 6-hourly basis: correlation between O_2 consumption and C content of the waste.
